# Encoding of frequency-modulation (FM) rates in human auditory cortex

**DOI:** 10.1038/srep18143

**Published:** 2015-12-14

**Authors:** Hidehiko Okamoto, Ryusuke Kakigi

**Affiliations:** 1 Department of Integrative Physiology, National Institute for Physiological Sciences, Okazaki, Japan; 2 Department of Physiological Sciences, School of Life Science, SOKENDAI (The Graduate University for Advanced Studies), Hayama, Japan

## Abstract

Frequency-modulated sounds play an important role in our daily social life. However, it currently remains unclear whether frequency modulation rates affect neural activity in the human auditory cortex. In the present study, using magnetoencephalography, we investigated the auditory evoked N1m and sustained field responses elicited by temporally repeated and superimposed frequency-modulated sweeps that were matched in the spectral domain, but differed in frequency modulation rates (1, 4, 16, and 64 octaves per sec). The results obtained demonstrated that the higher rate frequency-modulated sweeps elicited the smaller N1m and the larger sustained field responses. Frequency modulation rate had a significant impact on the human brain responses, thereby providing a key for disentangling a series of natural frequency-modulated sounds such as speech and music.

In a daily situation, we are continually exposed to natural sounds that change their spectral components over time in a sound-specific manner. Frequency modulation (FM) in human speech, bird songs, and animal vocalizations plays an essential role in species-specific communications. For example, previous studies reported that changing the direction of the third formant transition sweep within a human voiced sound from upward to downward turned the speech perception from “ba” to “ga”^1^,^2^. Deficits in the processing of FM sweeps in childhood have been shown to lead to language learning impairments^3^ and the persistence of stuttering^4^. Moreover, impairments in FM sweep processing have been suggested to cause reading disabilities, which are not directly related to hearing ability^5^,^6^. The ability of humans to identify FM rates plays an important role in our daily social life; however, the neural mechanisms underlying FM encoding remains elusive in the human auditory cortex.

Sound waves that enter the cochlea vibrate specific portions of the basilar membrane that correspond to their spectral components. Vibrations of the basilar membrane are systematically converted into tonotopic representations of neural activity^7^,^8^,^9^. Previous neuroimaging studies revealed that the tonotopic maps of neural activity elicited by pure tones were preserved in the human auditory cortex using magnetoencephalography^10^,^11^ (MEG), functional magnetic resonance imaging (fMRI)^12^,^13^, and positron emission tomography^14^. The neural processing of FM sweeps needs to simultaneously decode fine frequency structures at a single time point and track their temporal changes over time^15^,^16^,^17^,^18^. In marmosets, FM sweeps were shown to activate the primary auditory cortex more strongly than pure tones^19^. Moreover, FM sweeps in cats more dominantly activated non-primary auditory areas than the primary auditory cortex in a modulation rate-dependent manner^20^,^21^,^22^; however, the effect of the FM rates on the neural activity remains elusive in the human auditory cortex.

The aim of the present study was to investigate the auditory evoked responses elicited by FM sweeps with different rates using MEG. The N1m amplitude is known to depend on the frequency components of the test sound signal; higher frequency sounds have been shown to elicit smaller N1m responses^23^ at shorter latencies^24^. Moreover, the neural generator of the auditory evoked response elicited by a low rate FM sweep demonstrated a tonotopic gradient in medial–lateral and anterior–posterior directions in the human auditory cortex^25^. It is crucial to use FM sweeps as test stimuli that are matched with respect to the spectral characteristics. Therefore, in the present study we investigated the auditory evoked responses elicited by temporally repeated and superimposed FM sweeps (Figs 1 and 2). This experimental design allowed us to measure the brain activity elicited by FM sweeps that were matched in the spectral domain, but differed in the frequency modulation rates.

## Results

A sufficient number of trials could be averaged for each condition after artefact rejection (mean ± standard deviation: FM_01 = 193.6 ± 8.4; FM_04 = 192.9 ± 8.0; FM_16 = 192.7 ± 8.8; FM_64 = 193.5 ± 7.7) and clear auditory evoked N1m and sustained field (SF) responses were obtained in each FM rate condition (cf. Fig. 3). The goodness-of-fits of the underlying dipolar source models for the averaged MEG waveforms of all the sensors was 95.5 ± 2.4% (mean ± standard deviation) in the N1m responses and 95.6 ± 3.2% in the SF responses. The mean source locations of the N1m and SF responses are displayed in Fig. 4. The centers of the estimated source locations significantly differed between the N1m and SF responses. The SF source location was located more medially and anterior than the N1m response in the both hemispheres as shown in the previous studies^26^,^27^.

The time courses of the N1m source strengths (from −200 to +350 msec) and SF source strengths (+450 to 1000 msec) grand-averaged across all participants (N = 14) are displayed in Fig. 5. The clear N1m response around the 100 msec latency and the stable SF between 500 and 1000 msec are clearly shown. The N1m responses had larger source strengths in the lower rate FM sweeps than in the higher rate FM sweeps. In contrast, the higher rate FM sweep conditions demonstrated larger SF source strengths than the lower rate FM sweep conditions.

Figure 6 shows the mean N1m source strengths and latencies in each FM rate condition together with the corresponding 95% confidence intervals obtained by boot-strap resampling tests (iteration = 100 000). A two-way repeated-measures analysis of variance (ANOVA) applied to the N1m source strength revealed significant main effects for FM-RATE (*F*_(3, 39)_ = 28.02, *p* < 0.001) and HEMISPHERE (*F*_(1, 13)_ = 7.23, *p* < 0.02), but no significant interaction between them. The post hoc multi-comparison revealed significant differences between FM_01 and FM_04 (*t*_(27)_ = 3.35, *p* < 0.02), FM_01 and FM_16 (*t*_(27)_ = 8.98, *p* < 0.001), FM_01 and FM_64 (*t*_(27)_ = 7.20, *p* < 0.001), FM_04 and FM_16 (*t*_(27)_ = 6.85, *p* < 0.001), and FM_04 and FM_64 (*t*_(27)_ = 5.72, *p* < 0.001). The N1m source strength gradually decreased with an increase in the FM sweep rate. A two-way repeated-measures ANOVA applied to the N1m latency resulted in a significant main effect for the FM-RATE (*F*_(3, 39)_ = 12.84, *p* < 0.001). However, there was neither a significant main effect of HEMISPHERE nor a significant interaction between FM-RATE and HEMISPHERE. The post hoc multi-comparison revealed significant differences between FM_01 and FM_16 (*t*_(27)_ = −3.40, *p* < 0.02), FM_01 and FM_64 (*t*_(27)_ = −4.49, *p* < 0.001), FM_04 and FM_64 (*t*_(27)_ = −4.28, *p* < 0.002), and FM_16 and FM_64 (*t*_(27)_ = −3.47, *p* < 0.02). The N1m latency gradually increased with an increase in the FM sweep rate.

Figure 7 shows the mean SF source strengths in each condition together with the corresponding 95% confidence intervals obtained by boot-strap resampling tests (iteration = 100 000). A two-way repeated-measures ANOVA applied to the SF source strength revealed a significant main effect for FM-RATE (*F*_(3, 39)_ = 6.67, *p* < 0.02). However, there was neither a significant main effect of HEMISPHERE nor a significant interaction between FM-RATE and HEMISPHERE. The post hoc multi-comparison revealed significant differences between FM_01 and FM_16 (*t*_(27)_ = −3.52, *p* < 0.01), FM_01 and FM_64 (*t*_(27)_ = −4.26, *p* < 0.002), FM_04 and FM_16 (*t*_(27)_ = −3.85, *p* < 0.004), and FM_04 and FM_64 (*t*_(27)_ = −3.38, *p* < 0.02). In contrast to the N1m source strength, higher rate FM sweeps resulted in larger SF source strengths.

## Discussion

**Figure 1:**
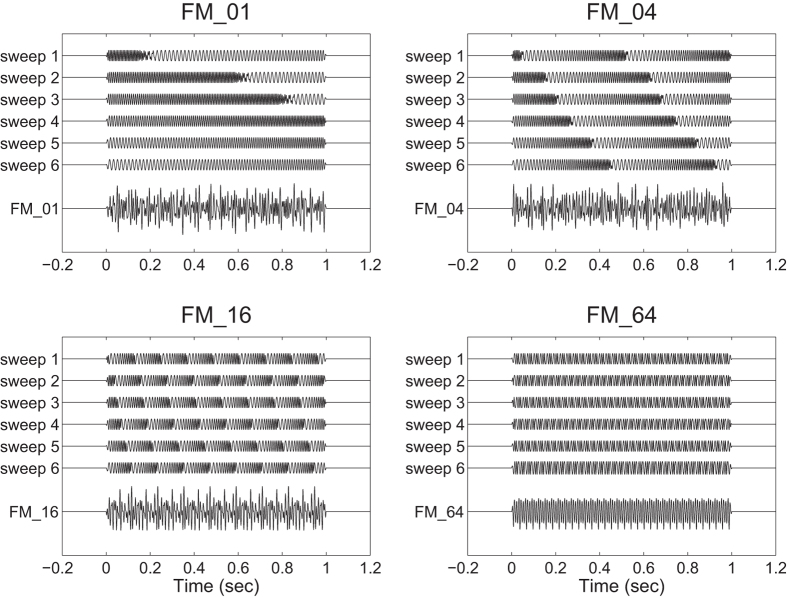
Schematic sound waveforms of test stimuli. Each test stimulus consisted of six temporally repeating frequency-modulated (FM) sweeps between 500 and 2000 Hz. The initial frequencies of the test stimuli were randomly selected between 500 and 2000 Hz and then frequency-modulated upward at 1 (FM_01), 4 (FM_04), 16 (FM_16), and 64 (FM_64) octaves per sec. Exemplary sound files are available in Additional files S1, S2. S3, and S4.

**Figure 2:**
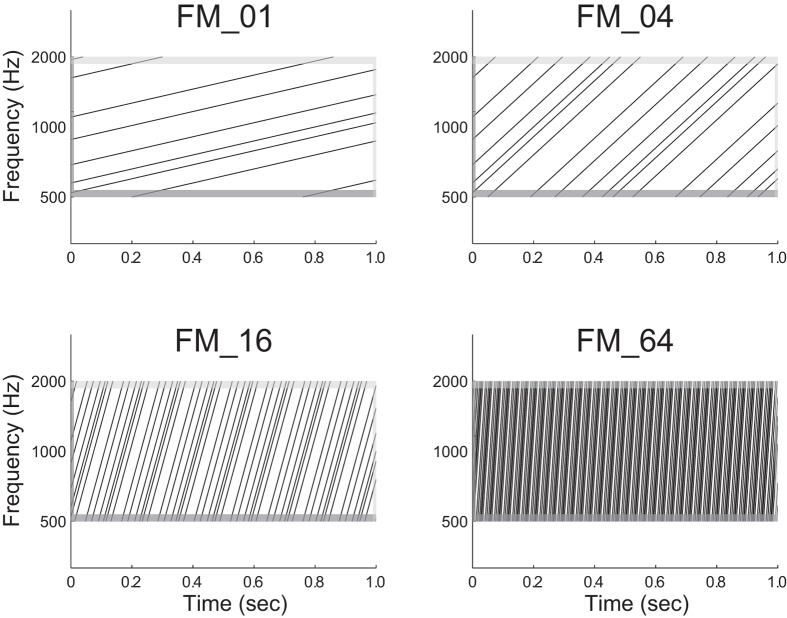
Schematic log-frequency spectrograms of test stimuli. All test stimuli consisted of six temporally repeating frequency-modulated (FM) tones travelling from 500 to 2000 Hz. The dark and light grey areas represent the linear rise- and fall-ramps of the sound signals, respectively. The spectral components at a single time point were similar between conditions. Exemplary sound files are available in Additional files S1, S2. S3, and S4.

In the present study, we investigated the auditory evoked N1m and SF responses elicited by temporally repeated and superimposed FM sweeps using MEG. The results obtained demonstrated that the N1m and SF source strengths were significantly influenced by the modulation rates of the test stimuli. The lowest FM rate test stimuli that changed their spectral components upward at the rate of 1 octave per sec (FM_01) caused the maximal N1m source strength, but, at the same time, caused the minimal SF source strength. Moreover, the N1m response elicited by the test stimuli showed a significant right hemispheric dominance. In the present study, we used temporally repeated and superimposed FM sweeps that consisted of six randomly chosen frequencies (Figs 1 and 2). Therefore, the test stimuli were balanced with respect to the spectral components. The results obtained suggest that the human auditory cortex is sensitive to the FM rates of the sound signals and plays an important role in the perception of complex natural sounds that change spectral information over time.

The N1m response is considered to reflect neural activity corresponding to the initial 20–40 msec part of the auditory signal^28^ in the lateral aspects of Heschl’ s gyrus and the temporal plane^29^,^30^,^31^. Within the initial 20 −40 msec of the test stimuli the higher rate FM sweeps would traverse broader areas within the tonotopic maps than the lower rate FM sweeps (cf. Fig. 1). If the neural activity elicited by FM sweeps merely represented the summation of activated neural areas in the tonotopic maps, the N1m responses elicited by the higher rate test stimuli would become larger than those elicited by lower rate test stimuli. However, the present results showed that the N1m responses elicited by the higher rate test stimuli were significantly smaller than those elicited by the lower rate test stimuli.

Previous studies also demonstrated that broader band-pass noise elicited smaller N1m responses than narrower band-pass noise^32^. Afferent neural inputs in the auditory pathway not only consist of excitatory connections, but also inhibitory neural mechanisms such as lateral inhibition^33^,^34^,^35^,^36^ and co-tuned inhibition^37^,^38^. Lateral inhibition is a neural concept in which excitatory neurons project not only an excitatory connection to neurons with the same receptive field, but also broadly tuned inhibitory neural connections to the surrounding neurons. The co-tuned excitatory and inhibitory neural model suggests that the frequency tuning curves of excitatory and inhibitory inputs are similar, whereas inhibitory inputs follow excitatory inputs after a short delay. These inhibitory neural mechanisms appear to contribute to improvements in frequency tuning in the auditory system^33^,^37^,^39^,^40^. In the present study the auditory neurons activated at a single time point may have suppressed surrounding neural activity within the tonotopic map. The surrounding neurons suppressed via lateral inhibition and/or co-tuned inhibition may have been activated shortly after by test stimuli with frequency components that were slightly upward modulated. Therefore, the smaller N1m responses elicited by the higher rate FM sweeps may reflect inhibitory neural networks within tonotopic maps in the human auditory system.

**Figure 3:**
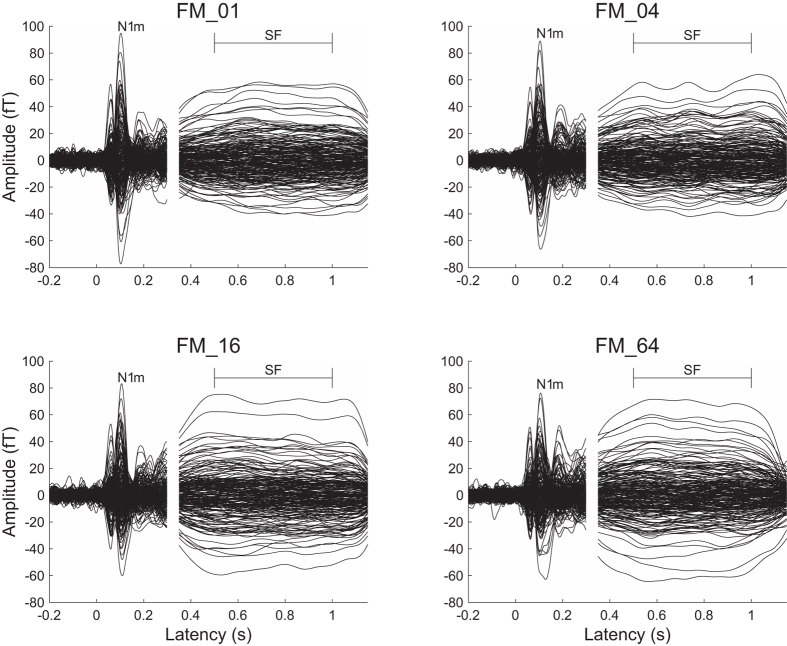
Auditory evoked N1m responses at 0.1 sec and sustained fields (SF: 0.5–1.0 sec) of one representative participant. The 30 Hz low-pass filtered magnetic fields for the N1m response are displayed between −0.2 and 0.3 sec and those 5 Hz low-pass filtered for the SF response were displayed between 0.35 and 1.15 sec.

**Figure 4:**
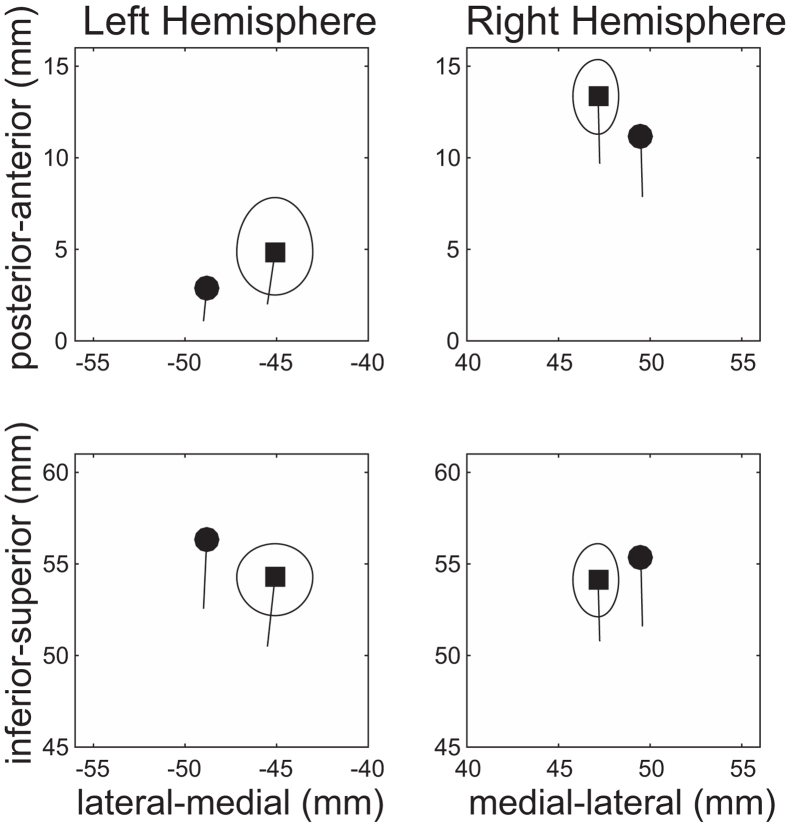
Grand averaged localization of N1m (filled circles) and sustained field (SF: filled squares) sources in the axial plane (medial-lateral direction vs. posterior-anterior direction, upper graph) and in the coronal plane (medial-lateral direction vs inferior-superior direction, lower graph). The solid line starting at each source location represents the mean orientation of the equivalent current dipole. The ellipses around SF source locations denote the 95% confidence intervals for the distance between N1m and SF sources.

**Figure 5:**
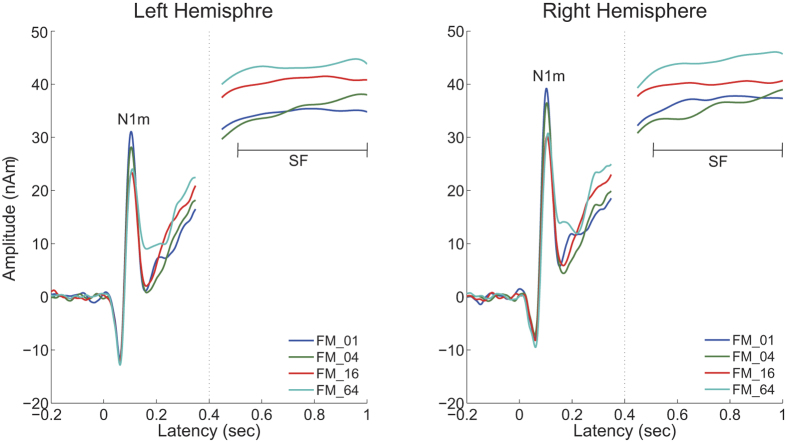
Source strength waveforms calculated at the N1m generator (−0.2 to 0.35 sec) and sustained field (SF) generator (0.45–1.0 sec) averaged across all participants (N = 14) in the left and right hemispheres. Each colored line represents each frequency modulation (FM) rate condition (see legends in the right bottom corner).

**Figure 6:**
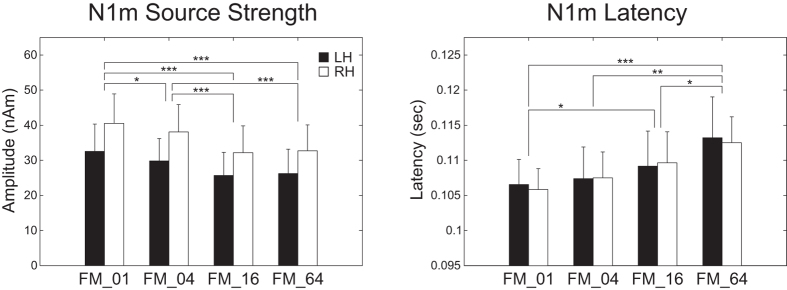
Group means (N = 14) of N1m source strengths (left panel) and latencies (right panel) under FM_01, FM_04, FM_16, and FM_64 conditions (from left to right), including error bars denoting 95% confidence intervals. Filled and open bars denote the N1m responses in the left hemisphere (LH) and right hemisphere (RH), respectively. (*p < 0.05, **p < 0.01, ***p < 0.001 [Bonferroni-corrected]).

**Figure 7:**
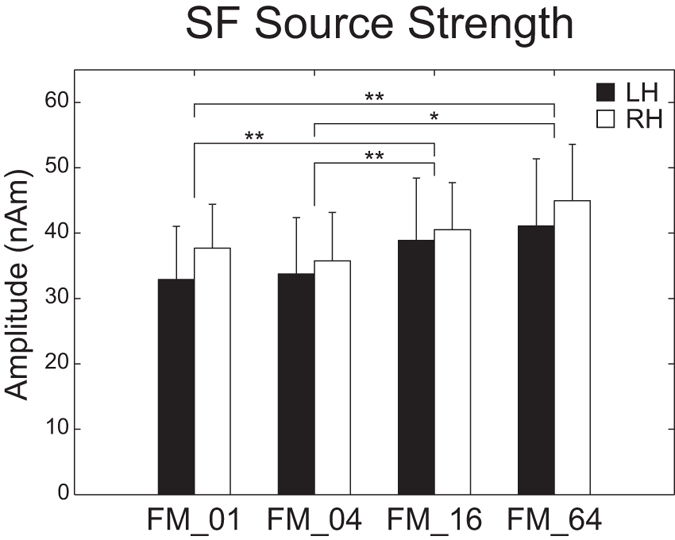
Group means (N = 14) of sustained field (SF) source strengths under FM_01, FM_04, FM_16, and FM_64 conditions (from left to right) including error bars denoting 95% confidence intervals. Filled and open bars denote the SF source strengths in the left hemisphere (LH) and right hemisphere (RH), respectively. (*p < 0.05, **p < 0.01, ***p < 0.001 [Bonferroni-corrected]).

The SF response is the DC-shift in magnetic fields originating in the auditory belt region^27^,^41^. We used a relatively long (1 sec) test stimulus and successfully obtained stable SF responses, as shown in Fig. 3. The SF appears to reflect the temporal regularity of the auditory signals. Gutschalk *et al.*^41^ demonstrated that regularly presented clicks elicited larger SF amplitudes than irregular clicks and also that higher click rates elicited larger SF amplitudes. Moreover, Keceli *et al.*^42^ measured the SF responses elicited by white noise and temporally repeated frozen noises at rates of 5, 10, 50, 200, and 500 Hz and showed that SF amplitudes were maximal when the noises were temporally repeated at rates of 10 and 50 Hz. The SF source strengths obtained in the present study were larger under the FM_16 and FM_64 conditions than under the FM_01 and FM_04 conditions (Figs 3, 5 and 7). As shown in Figs 1 and 2, the test sound signals used in the present study contained the temporally repeated structure at rates of 0.5 Hz (FM_01), 2 Hz (FM_04), 8 Hz (FM_16), and 32 Hz (FM_64). Therefore, the SF source strengths elicited by the test stimuli in the present study appeared to represent the detection of the repeated segments of the sound signals, leading to the perception of sound periodicity.

The present results showed significantly larger N1m source strengths elicited by the test stimuli in the right hemisphere than in the left. Functional hemispheric lateralization of the human brain is often observed in the auditory processing of complex sound stimuli such as language^26^,^43^,^44^,^45^ and music^46^,^47^. However, the hemispheric asymmetry of neural activity in the human auditory cortex does not appear to be limited to complex natural sounds, but rather originates from the basic neural processing of elementary sound features. A previous MEG study demonstrated that the N1m responses elicited by spectral changes were larger in the right hemisphere than in the left, whereas those elicited by sound-envelope changes were larger in the left hemisphere than in the right^48^. This hemispheric asymmetry of neural activity in auditory processing may be explained by different integration time windows adopted in the left and right hemispheres. The right human auditory cortex appears to be specialized for the precise spectral processing of incoming sounds by applying longer integration time windows than those in the left^49^,^50^,^51^, leading to finer frequency tuning in the right hemisphere^52^. Previous studies on Mongolian gerbils^53^ and rats^54^ demonstrated that lesions in the right auditory cortex markedly impaired the discrimination of FM sweeps, whereas left auditory cortex lesions did not cause such an impairment. In humans, epileptic patients who underwent surgical resection from the right temporal lobe showed significant impairments in judging the direction of pitch change, whereas those who underwent left temporal lobe resection performed pitch direction judgements normally^55^. The right hemispheric dominancy of the N1m response observed in the present study also supported the hypothesis that the right hemisphere plays a major role in the neural encoding of auditory signals with spectral components that change over time.

In contrast to the present results, previous studies using fMRI^56^,^57^ found no significant effect of the FM rate on brain activity; however, one study^57^ reported the left hemispheric dominance of neural activity elicited by FM sounds. These differences appear to have originated from several methodological differences. FMRI measures hemodynamic responses to brain activity with a temporal resolution of a few seconds^58^, whereas MEG records magnetic fields originating from electrical currents in the human brain with a temporal resolution in the order of milliseconds. Therefore, the findings of fMRI studies appeared to include the summation of neural activity corresponding to N1m and SF responses. The decrement in the N1m amplitude and increment in the SF amplitude with increases in the FM rate may cancel each other out in the hemodynamic response. We used temporally repeated and superimposed FM sweeps traveling in an upward direction only as test sound stimuli, whereas Hsieh *et al.*^56^ used super-imposed FM sweeps containing five FM tones with a 1/3 octave spacing that were frequency modulated in an up- or downward direction at a rate of either 0.83 or 3.3 octaves per sec, and Joanisse and DeSouza^57^ used iterative rippled noises that were up- or downward frequency-modulated at a high (20 octaves/sec) or low (10 octaves/sec) rate with a duration of 50 or 100 msec, respectively. Therefore, in contrast to the present results, the findings of previous fMRI studies showed that the durations of FM stimuli varied between the different FM rate conditions used and neural activity elicited by up- and downward FM stimuli was combined in order to examine the FM rate effect. We used inharmonic FM sweeps that consisted of six random frequencies; however, Joanisse and DeSouza^57^ used speech-like harmonic sounds as test stimuli, which have been shown to elicit stronger neural activation in the left human auditory cortex^59^,^60^. Moreover, the iterative rippled noise stimuli used in their study^57^ yielded rapid envelope changes during stimulus presentation, which are known to produce stronger neural activity in the left hemisphere than in the right^48^,^51^. Joanisse and DeSouza^57^ also found that brain activation was greater in the left auditory cortex for the control iterative rippled noise that was not frequency-modulated. These variations in the physical properties of sound stimuli appeared to contribute to the different effects of FM rates on brain activity and the hemispheric lateralization of neural activity elicited by FM sounds.

In conclusion, using appropriately and carefully constructed auditory stimuli, we herein clearly demonstrated that the rate of frequency-modulated sweeps significantly influenced the neural activity in the human auditory cortex. The sensitivity of the auditory evoked responses elicited by the FM sweeps with different modulation rates appears to contribute to decoding complex natural auditory scenes, which contain several spectral components that change over time with different rates and directions.

## Methods

### Participants

Fifteen healthy individuals participated in the present study. All participants were right-handed (assessed via Edinburgh Handedness Inventory^61^) their hearing thresholds were within norms for the frequency range of 250–8000 Hz, as tested by means of clinical pure tone audiometry (AA-71, RION Co., LTD, Tokyo, Japan). One participant was excluded from further analyses because of the poor quality of magnetoencephalography data. Thus, the data of fourteen participants (9 females; mean ± standard deviation: 25.1 ± 7.5 years) were analyzed. They were fully informed about the study and gave written informed consent for their participation in accordance with procedures approved by the Ethics Commission of the National Institute for Physiological Sciences. Therefore, the study conformed to The Code of Ethics of the World Medical Association (Declaration of Helsinki).

### Stimuli and experimental design

Test stimuli were diotically presented and had a duration of 1000 msec with 10-msec linear onset and offset ramps (sampling rate: 48000 Hz). They were composed from six FM tones that traversed the frequency range (500–2000 Hz) in the upward direction, as schematically represented in Figs 1 and 2 [listen to Supplemental Audio S1 (FM_01), S2 (FM_04), S3 (FM_16), and S4 (FM_64) (supplemental data for this study are available online)]. Six initial frequencies were randomly chosen between 500 and 2000 Hz in a logarithmic scale. Then these six frequencies were modulated at sweep rates of 1 (FM_01), 4 (FM_04), 16 (FM_16), and 64 octaves (FM_64) per sec. Therefore, the initial frequencies were identical between the FM_01, FM_04, FM_16, and FM_64 conditions. We prepared 200 distinct FM sweeps in each FM rate condition. The FM tones in the FM_01, FM_04, FM_16, and FM_64 had 100-, 25-, 6.25-, and 1.56-msec linear rise ramps starting at 500 Hz and linear fall ramps ending at 2000 Hz, respectively (cf. Fig. 2). The onsets of the rise and fall ramps occurred simultaneously in order to avoid the sound envelope change. All test stimuli were adjusted to have an identical sound pressure level and were pseudo-randomly presented through plastic tubes that were 1.5 m in length and earpieces fit to the participant’s ears (E-A-Rtone 3A, Aero Company, Indianapolis, IN). Before starting data acquisition by MEG, each participant’s hearing threshold for an FM_01 was individually determined for each ear. During the MEG recording session, test stimuli were presented at an intensity of 50 dB above individual sensation levels. Sound onset asynchrony between subsequent test stimuli was randomized between 2 and 3 sec. In order to keep the participants alert during the MEG measurements, a self-chosen silent movie was presented. At the end of the measurements, questions regarding the content of the movie were asked to ensure that the participants had paid attention to the silent movie.

### Data acquisition and analysis

Auditory evoked fields were measured with a helmet-shaped 204-channel whole head planar-type gradiometer (Vector-view, ELEKTA, Neuromag, Helsinki, Finland) in a silent, magnetically shielded room. Signals were passed through a 200 Hz low-pass filter and digitally recorded (sampling rate = 600 Hz). The magnetic fields evoked by the test stimuli were averaged selectively for each FM rate condition, starting 200 msec prior to the sound onset, and ending 200 msec after the sound offset. Epochs containing amplitude changes greater than 3 pico-Tesla/cm were discarded as artefact-contaminated epochs.

The source locations and orientations of the auditory evoked fields were estimated using BESA software (BESA Research 5.3.7, BESA GmbH, Germany). The origins of the locations and orientations of the equivalent dipolar sources of N1m and SF responses were determined in a Cartesian coordinate system using three-shell spherical head model with the medial–lateral axis connecting the pre-auricular points of both ears. The posterior–anterior axis ran through the nasion perpendicular to the medial–lateral axis, and the inferior–superior axis ran perpendicularly to the medial–lateral and posterior–anterior axes.

In order to analyze the N1m component, the grand-averaged magnetic field signals elicited by all the test stimuli after artefact rejection were 30 Hz low-pass filtered (zero-phase shift Butterworth filter, 24 dB/oct), and the baseline was corrected by subtracting the mean amplitude of the 200 msec pre-stimulus interval. The peak N1m response was initially identified as the maximal root-mean square value of the global field power of all the sensors approximately 100 msec after the test stimulus onset. Source locations and orientations were then individually estimated using all the sensors based on the 10-msec time window around the N1m peak with the single equivalent current dipole model for each participant and each hemisphere. The estimated sources, which were fixed in location and orientation for each hemisphere in each participant, served as a spatial filter^62^ during the calculation of the source strength for all time points for each FM rate condition. The calculated source strength waveforms were used to obtain the maximal N1m source strength and N1m latency in each participant, each hemisphere, and each FM condition in the time range between 80 and 140 msec.

In order to analyze the auditory evoked SF responses, the grand-averaged magnetic field responses elicited by all the test stimuli after the artefact rejection were 5-Hz low-pass filtered (zero-phase shift Butterworth filter, 24 dB/oct) and the baseline was corrected relative to a 200 msec pre-stimulus interval. The fixed source locations and orientations were approximated between 500 and 1000 msec for the grand-averaged MEG waveforms in the same way as the N1m response. The estimated source for each participant in each hemisphere was fixed in its location and orientation, and the mean source strength between 500 and 1000 msec was calculated for each FM rate condition and used for further analyses. The N1m and SF source strengths were evaluated separately by means of the two-way repeated-measures ANOVA using HEMISPHERE (Left vs. Right) and FM-RATE (FM_01, FM_04, FM_16, and FM_64) as factors. The N1m latencies were similarly evaluated by means of repeated-measures ANOVA. In this study, the *p* values provided for repeated-measures ANOVA results were Greenhouse–Geisser corrected, and Bonferroni corrected paired t-tests were performed for post hoc multi-comparisons.

## Additional Information

**How to cite this article**: Okamoto, H. and Kakigi, R. Encoding of frequency-modulation (FM) rates in human auditory cortex. *Sci. Rep.* **5**, 18143; doi: 10.1038/srep18143 (2015).

## Supplementary Material

Supplementary Information

Supplementary S1

Supplementary S2

Supplementary S3

Supplementary S4
