# Supplementary material for: Encoding of frequency-modulation (FM) rates in human auditory cortex
Source: Sci Rep. 2015 Dec 14;5:18143. doi: 10.1038/srep18143 (PMC4677350; doi:10.1038/srep18143)
Supplement: Supplementary Information [file srep18143-s1.pdf]

Encoding of frequency-modulation (FM) rates in human auditory cortex

Hidehiko Okamoto, Ryusuke Kakigi

Supplementary Material File Names

S1. Example for FM_01.wav

S2. Example for FM_04.wav

S3. Example for FM_16.wav

S4. Example for FM_64.wav

Supplementary Material Legends

SI 1. An exemplary sound representing FM_01 as shown in Figures 1 and 2.

SI 2. An exemplary sound representing FM_04 as shown in Figures 1 and 2.

SI 3. An exemplary sound representing FM_16 as shown in Figures 1 and 2.

SI 4. An exemplary sound representing FM_64 as shown in Figures 1 and 2.
